# Mind the gap: connexins and pannexins in platelet function

**DOI:** 10.1080/09537104.2021.1902971

**Published:** 2021-04-05

**Authors:** Kirk a Taylor, Gemma Little, Jonathan M. Gibbins

**Affiliations:** 1 Institute for Cardiovascular and Metabolic Research, University of Reading, Reading, UK; 2 National Heart and Lung Institute, Imperial College London, London, UK

**Keywords:** Platelets; Ion Channel; Connexin; Pannexin-1; Gap junction

## Abstract

Connexins are a family of gap junction forming proteins widely expressed by mammalian cells. They assemble into hexameric hemichannels, which can either function independently or dock with opposing hemichannels on apposite cells, forming a gap junction. Pannexins are structurally related to the connexins but extensive glycosylation of these channels prevents docking to form gap junctions and they function as membrane channels. Platelets express pannexin-1 and several connexin family members (Cx37, Cx40 and Cx62). These channels are permeable to molecules up to 1,000 Daltons in molecular mass and functional studies demonstrate their role in non-vesicular ATP release. Channel activation is regulated by (patho)physiological stimuli, such as mechanical stimulation, making them attractive potential drug targets for the management of arterial thrombosis. This review explores the structure and function of platelet pannexin-1 and connexins, the mechanisms by which they are gated and their therapeutic potential.

## Introduction

Platelets circulate in a complex environment, interacting with other blood cells and the vessel wall. Vascular injury or rupture of atherosclerotic plaques expose thrombogenic molecules (e.g., vWF and collagen) that initiate platelet recruitment, activation and thrombus formation, resulting in hemostasis or thrombosis, respectively. The platelet response to injury is rapid and proportionate, orchestrated by complex interplay of activatory and inhibitory signals [1]. Conversely, arterial thrombosis arises from pathological platelet activation and leads to myocardial infarction and stroke. The discovery of platelet connexin (Cx) gap junction proteins and a structurally related pannexin-1 (Panx1) channel on platelets may provide previously unrecognized mechanisms for communication between platelets to regulate the thrombotic response. This review explores recent developments in the structure and function of platelet connexins and Panx1, the potential roles that these proteins may play in platelet-related diseases and their prospects as antiplatelet targets.

## Structure and Function of Connexins and Pannexins

**Figure 1.**
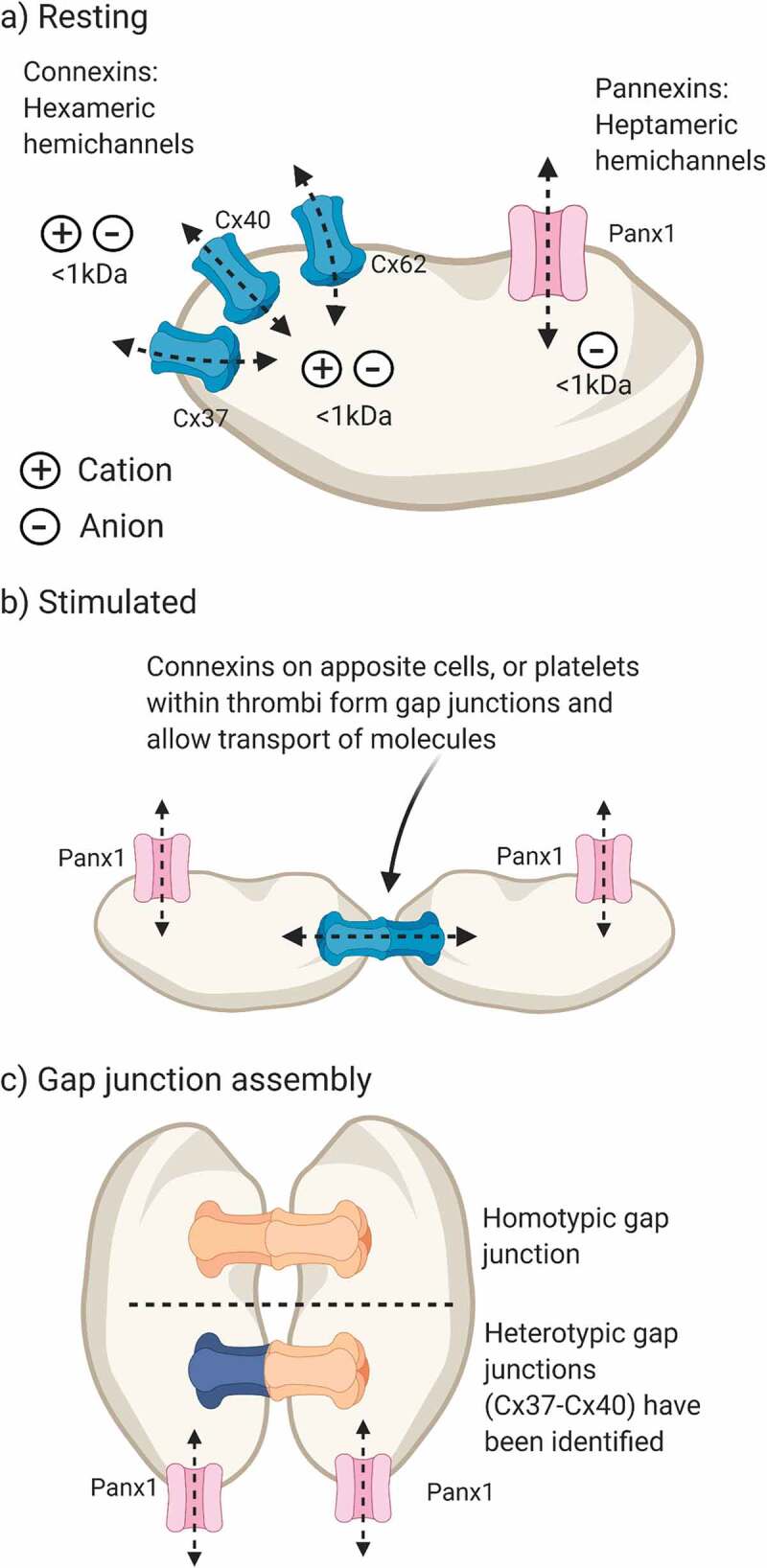
**Expression and assembly of platelet connexins and pannexin-1**. (a) Platelets express Cx37, Cx40 and Cx62 and pannexin-1 (Panx1). Connexins form nonselective hexameric hemichannels and pannexin-1 forms heptameric anion-selective channels that are detected at the plasma membrane. (b) Docking of two apposite hemichannels forms a gap junction that facilitates intercellular communication. (c) Gap junctions form between two hemichannels by either homotypic (e.g., Cx37:Cx37) or heterotypic (e.g., Cx37:Cx40) assembly, which may influence gap junction permeability. The molecular identity of platelet gap junctions and whether they are homo/heterotypic has not been studied. Panx1 cannot form gap junctions due to glycosylation on its second extracellular loop. Created in BioRender

Connexins have been studied widely and members of this family of 21 proteins [2] play critical roles in regulating signal transduction and propagating calcium waves in a range of tissues. Connexin gap junctions ensure coordinated responses by neighboring cells (reviewed in [3]) since connexin mediated intercellular signaling is limited only by the rate of diffusion of signal molecules conveyed by these channels. Connexins form a broad family of proteins and are denoted by their molecular mass (i.e., Cx32 displays an apparent molecular mass of 32 kDa when analyzed by SDS-PAGE). Connexin monomers assemble into hexameric hemichannels and are trafficked to the plasma membrane where docking of two hemichannels on apposite cells forms a gap junction, allowing intercellular flow of molecules up to 1000 Daltons in size. Connexin hemichannels may be formed by a single member of the connexin family (homomeric) or from a mixture of family members (heteromeric) [4]. Docking of homomeric or heteromeric hemichannels, gives rise to assembly of homotypic and heterotypic gap junctions, respectively (Summarized in Figure 1). Intermixing of connexins in this way has been shown to influence the nature of intercellular signal transduction; for example, Cx26:Cx37 heterotypic gap junctions have enhanced permeability to cGMP over cAMP, compared with Cx37 homotypic junctions [4]. The functional significance of potential connexin intermixing in platelets is not understood, although it is interesting to note the substantial and similar impact of the inhibition or deletion of each of Cx37, 40 and 62, which may suggest that platelet connexins may collaborate in these cells [5–7]. Connexins also function as hemichannels at the plasma membrane, providing a route for release of cytosolic signaling molecules, such as ATP [8]. Hemichannel and gap junction modalities may provide distinct roles for connexins in freely circulating platelets and within thrombi, respectively.

Pannexins were first reported in 2000 by Panchina and colleagues, during a genetic screen for invertebrate gap junction homologues [9]. This family of three proteins displays distinct expression patterns whereby Panx1 is ubiquitously expressed, pannexin-2 is restricted to the brain and central nervous system and pannexin-3 is predominantly found in bone and skin cells [10]. Early studies of Panx1 suggested that these proteins assemble into hexameric channels before becoming glycosylated and trafficked to the plasma membrane [11]. However, this classical view of hexameric channels has been challenged by two independent cryo-EM studies of the Panx1 structure, which report that seven Panx1 monomers assemble to form heptameric channels [12,13]. These channels diverge further from connexins as glycosylation of the second extracellular loop at N254 prevents docking of apposite Panx1 channels [14]. This was tested by two-electrode voltage clamp measurements of *Xenopus laevis* oocytes expressing Panx1. When the glycosidase PNGaseF was applied to the bath solution, N-linked glycans on Panx1 were broken down and a junctional conductance could be recorded [11]. Under physiological conditions Panx1 does not form gap junctions[15], rather, these proteins form anion-selective ion channels at the plasma membrane (Figure 1) [16].

## Expression and Localization of Platelet Pannexin-1 and Connexins

Platelet proteomic and transcriptomic analyses report expression of Panx1 and significant levels of Cx32, Cx37, Cx40 and Cx62 [17–20]. Panx1 is the sole pannexin expressed by platelets and qPCR analysis of platelet mRNA demonstrated that Panx1 may be expressed at similar levels to the ATP-gated ion channel P2X1 [21]. Protein expression, biotinylation and biochemical studies suggest that platelet Panx1 is glycosylated and the majority of these channels are expressed at the plasma membrane, where they become activated downstream of agonist-evoked platelet activation [21]. Molica and colleagues reported a functional defect in subjects that have a single nucleotide polymorphism of C400A, leading to reduced collagen-evoked platelet aggregation responses [22].

Of the 16 connexins screened from blood cells and megakaryocytes, notable levels of Cx37, Cx40 and Cx62 mRNA were detected from cultured megakaryocytes [18]. Expression of Cx37 and Cx40 has been confirmed by Western blotting, immunocytochemistry and flow cytometry [5,6,18]. More recently, Cx62 was identified in human platelets, where it was shown to reside intracellularly before translocating to the plasma membrane upon stimulation by the thromboxane A2 mimetic U46619 [7]. People carrying a single nucleotide polymorphism P319S in the Cx37 gene display modestly enhanced platelet activation, suggesting a role for Cx37 in regulating platelet activation [5]. To date, platelet connexins have been shown to function both as hemichannels and gap junctions [5–7,18]. Of the platelet connexins, evidence suggests that whilst Cx37 and Cx40 cannot form heteromeric structures, they are able to assemble in a heterotypic manner between apposite cells [23]. Clot retraction is a coordinated process whereby microtubules contract to facilitate wound healing [24]. Interestingly, selective peptide inhibitors of either Cx37, Cx40 or Cx62 inhibit clot retraction, suggesting that there may be a role for gap junctions in the coordination of this process [6,7,18]. Connexin intermixing has not been studied in platelets but this could serve to regulate platelet interactions within a thrombus or with other cell types.

Functional roles for Panx1 and platelet connexins have been demonstrated *in vitro* and *in vivo*. Conflicting reports indicate that global knockout of Cx37 reduces bleeding time [5], whilst administration of a Cx37 blocking peptide decreases thrombus formation *in vivo* [18]. The underlying differences between these reports remain unclear and further studies with platelet-specific deletion of Cx37 are required. Furthermore, Cx40^−/-^ mice have increased tail bleeding times, suggesting a regulatory role for this connexin during platelet activation [6]. Cx62 has been shown to contribute to thrombus formation *in vivo* as thrombi were significantly smaller in mice treated with a selective inhibitory peptide [7]. A common platelet phenotype was observed with each connexin knockout mouse whereby fibrinogen binding, *P*-selectin exposure and ATP release were all reduced [6,7]. *In vivo* studies demonstrate that Panx1 contributes to both hemostasis and thrombosis with significantly elevated tail bleeding times and reduced mortality in a thromboembolic model in Panx1^−/-^ mice [25]. Extensive evidence therefore supports the notion that connexins, specifically Cx37^5^[18], 40^6^ and 62^7^, and Panx1^21^ [25,26], perform important regulatory roles in platelet function in hemostasis and thrombosis.

## What Messages are Conveyed by Connexins and Pannexins

Connexins and Panx1 contribute to platelet function *in vivo* and may represent potential antithrombotic targets. These proteins form large pores but the precise nature of the signals conveyed by platelet connexins and/or Panx1 remains unclear. Functional studies have tended to focus on the loss or transfer of cytosolic calcein, a 622 Dalton anionic fluorescent dye, in flow cytometry and FRAP (fluorescence recovery after photobleaching) assays [7,21]. These channels are also known to represent pathways for non-vesicular release of cytosolic ATP [27,28]. ATP release through Panx1 and connexin hemichannels has been demonstrated using ATP-dependent luciferin-luciferase assays in platelet suspensions [6,7,18,21]. To date, transfer of other molecules, such as ADP, cyclic nucleotides, ions (e.g., Ca^2+^^2^, Cl^−^, Zn^2+^) and inositol-1,4,5-trisphosphate (IP3), has not been demonstrated in platelets or megakaryocytes. It is tempting to speculate that transfer of IP3 *via* gap junctions within a thrombus, akin to that within cardiac muscle[29], may serve to coordinate and propagate platelet calcium waves within a growing thrombus.

## Regulation of Pannexin and Connexin Channels

Connexin and pannexin channels and junctions must be appropriately regulated to prevent leakage of cytosolic content (e.g., ATP) and influx of extracellular ions (e.g., Ca^2+^), which would disrupt various aspects of cellular homeostasis. To this end, multiple regulatory mechanisms have evolved to control gating of pannexin and connexin channels and ultimately gap junction formation. Such mechanisms include post-translational modifications (PTMs), mechanical stimulation, ischemia, channel gating by extracellular ions and enzymatic cleavage [27,30–35], and are explored herein.

### *Phosphorylation (**Figure 2A*)

Regulation of platelet connexins and Panx1 by PTMs presents a mechanism for the efficient switching on/off of these channels in response to environmental cues. In the case of Panx1, several putative phosphorylation sites have been reported for Src Family Kinases (SFKs), protein Kinase A (PKA) and Protein Kinase C (PKC) [31,36,37]. Collagen-evoked platelet activation leads to Panx1 phosphorylation at Y308, which is inhibited by preincubation with the SFK inhibitor PP2 [25]. The functional relevance of predicted PKA and PKC sites on Panx1 are not yet clear. Regulation of platelet Cx37 and Cx40 by phosphorylation is unknown, although UniProt annotations suggest that these channels have several predicted serine, threonine and tyrosine phosphorylation sites. Related Cx43 channels are broadly expressed and deletion of this gene is embryonic lethal [35]. A detailed review of Cx43 phosphorylation [38] highlights 19 conserved kinase recognition motifs in the C terminus, and discusses how changes in Cx43 phosphorylation lead to alteration of hemichannel and gap junction permeability and electrophysiological properties. Cx37 has been shown to be negatively regulated by PKC in rat insulinoma cells, where PKC inhibition increased the open state probability [30]. A potential link between Cx62 and PKA was recently reported; platelets incubated with a selective Cx62 inhibitory peptide had increased levels of PKA-mediated VASP phosphorylation, which occurs in a cAMP-independent manner [7]. It is currently unclear whether this implicates PKA in the phosphorylation and regulation of Cx62 function, or that Cx62 is able to induce inhibitory signaling pathways that involve PKA. Further studies are required to fully understand the functional significance of phosphorylation-mediated regulation of platelet connexins and Panx1.

**Figure 2.**
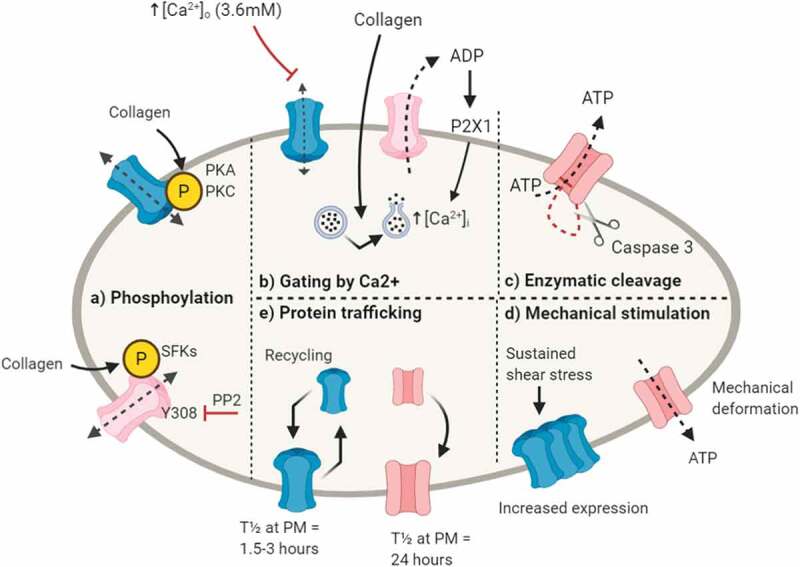
**Proposed mechanisms for regulation of platelet connexins and pannexin-1**. (a) *Phosphorylation-* Pannexin-1 (Panx1) is phosphorylated by Src Family Kinases (SFKs) at Y308, leading to channel opening. Platelet connexins have predicted phosphorylation protein kinase C (PKC) sites and Cx62 may interact with protein kinase A (PKA). (b) *Gating by Ca+-* Connexin hemichannels are open in the absence of extracellular Ca^2+^ ([Ca^2+^_o_) and close when [Ca^2+^]_o_ rises to 2–4 mM. Panx1 channels open in response to a rise of intracellular Ca^2+^ ([Ca^2+^]_i_) that leads to ATP release and stimulation of P2X1 on platelets. (c) *Enzymatic cleavage*- The intracellular C-terminus of Panx1 contains a zymogen site that is cleaved by caspase-3, removing an auto inhibitory domain, facilitating channel activation and ATP release. (d) *Mechanical stimulation*- Mechanical deformation and elevated shear stress have been shown to stimulate Panx1 channels. Studies of cardiac muscle report increased expression of Cx43 following sustained exposure to shear stress. (e) *Protein trafficking*- Connexins are expressed at the plasma membrane for relatively short periods (half-life (T_1/2_) = 1.5–3 hours) and are trafficked and recycling in response to cellular activation. Panx1 is stably expressed at the plasma membrane with a T_1/2_ of approximately 24 hours. Created in BioRender

### *Gating by Ca^2+^* (*Figure 2B*)

Gating of connexin hemichannels by extracellular ([Ca^2+^]_0_) was demonstrated by atomic force microscopy studies in HeLa cells expressing recombinant Cx40. In this study hemichannels were open in Ca+ free conditions but closed when [Ca^2+^]_0_ was elevated to 3.6 mM [39]. Molecular dynamic simulations based on Cx26 suggest that a [Ca+]_0_ sensing motif within the hemichannel pore is responsible for channel closure and this motif is conserved between connexin family members [32]. Whilst, these studies suggest that physiological levels of [Ca^2+^] markedly reduce the open probability of connexin hemichannels, it is not clear whether this phenomenon can be overcome by other regulatory mechanisms, such as phosphorylation or mechanical stimulation. Given that connexins contribute to platelet activation in flow cytometric studies with physiological levels of [Ca^2+^]_0_ [5–7,18], where hemichannels are predicted to be closed, it is possible that this inhibition could be overcome by direct stimulation of the connexin by phosphorylation or mechanical forces. Panx1 channels do not appear to be directly affected by fluctuations of [Ca^2+^]_o_ [40], rather elevation of intracellular Ca+ ([Ca^2+^]_o_) leads to the activation of Panx1, *via* an unknown mechanism [41]. Activation of platelet Panx1 was proposed to be mediated by collagen-evoked [Ca^2+^]_o_+ calcium signaling, leading to ATP release and amplification of the aggregation response [21]. Further studies are required to unravel the mechanisms that govern Ca+-mediated gating of platelet Pannexin and Connexin channels.

### *Enzymatic Cleavage (**Figure 2C*)

Structural studies of Panx1 indicate that the cytoplasmic C-terminus becomes inserted into the pore region, serving as an autoinhibitory mechanism. Furthermore, this region has a zymogen site that is cleaved by caspase 3. Cleavage of the C-terminus leads to a terminal open state that is thought to release ATP as an apoptotic “find-me” signal [33,42]. These findings suggest that Panx1 may exist in multiple open states to respond to different stimuli. Indeed, direct stimulation of Panx1 by extracellular K^+^ led to channel activation with conductance of 500ps, whereas voltage-dependent activation of the channel led to a lower 50ps conductance that was not associated with ATP release [43]. Such regulation may enable Panx1 to release different cargos in response to different stimuli.

### *Mechanical Stimulation (**Figure 2D*)

Platelets and megakaryocytes express stretch-activated Piezo 1 [44], TRPM7 [45,46] and TRPV4 [47] cation channels. This could ultimately prove to be an important mechanism for shear-dependent platelet activation in the context of stenosed vessels with pathological shear rates. Intercellular transmission of Ca[2]+ signals has been reported during *in vitro* adhesion assays and displays an ADP-dependent component [48]. The pathway for this ADP release is unclear and may be mediated by dense granule release, Panx1 channels or connexin hemichannels. Notably, erythrocyte Panx1 channels were shown to release ATP in response to mechanical deformation [27]. Furthermore, sustained exposure to shear stress increased endothelial Cx40 expression [49]. Platelet adhesion to collagen under arteriolar shear rates (1000s^−1^) has been shown to be sensitive to connexin and Panx1 inhibitors [6,18,21].

### *Protein Trafficking (**Figure 2E*)

Protein trafficking represents an alternative mechanism for the regulation of pannexins and connexins. The half-life of connexin hemichannels at the plasma membrane is estimated to be between 1.5 and 5 h [50]. By comparison, heterologously expressed rat Panx1 channels in human embryonic kidney cells are stably expressed at the plasma membrane, with 45% of channels remaining after 24 h [11]. D-STORM imaging of platelet Cx62 demonstrated that these channels are increasingly associated with the plasma membrane following stimulation [7]. Further studies are required to evaluate the role of trafficking in the dynamic regulation of connexins in response to (patho)physiological stimuli.

## Therapeutic Potential

Connexins and Panx1 have been studied using the inhibitors carbenoxolone, probenecid, and spironolactone, which are also FDA-approved drugs for the management of tracheal ulcers, gouty arthritis and hypertension, respectively [18,21,27,51]. This raises the question as to whether these drugs could be repurposed to manage arterial thrombosis. One potential limitation to such an approach would be off-target effects upon other connexin family members or through inhibition of Panx1 on other cell types. For example, inhibitors of Cx40 in the circulation would not only target platelet function but also endothelial and cardiac cells that also express Cx40. It is worth noting, however, that Cx37^−/-^ mice do not have a cardiovascular phenotype and Cx40^−/-^ mice have reduced cardiac conduction velocity, leading to increased incidence of arrhythmias, suggesting that there may be some functional redundancy between family members [52–54]. Furthermore, it is worth considering that these drugs target other receptors, introducing the potential for undesired effects. However, these drugs have been in clinical use for several decades and therefore have established safety profiles. Interestingly, orally administered probenecid achieves plasma concentrations of ≈78 µM, which is sufficient to achieve inhibition of platelet Panx1 channels [21]. Probenecid has also been shown to enhance the effects of indomethacin and heparin, without increasing the plasma half-life of these drugs [55,56]. Pharmaco-epidemiological approaches could be employed to assess the relationship between administration of known gap junction or pannexin blockers on the incidence of cardiovascular diseases. However, this would require careful consideration of confounding factors as people diagnosed with gouty arthritis or hypertension may be predisposed to thrombotic diseases.

An alternative approach would be to develop small molecule inhibitors that target specific connexin or pannexin isoforms. Isotype specific inhibitory peptides that target the extracellular loops of connexins [7,57] and Panx1[58] have been employed successfully to study the function of these proteins *in vitro* and *in vivo*. These target sequences could therefore be used to inform the development of small molecule inhibitors to target these proteins on platelets and manage thrombosis.

*Ex vivo* analyses demonstrated that mouse platelets treated with probenecid, reduced murine platelet aggregation [25,51]. *In vitro* thrombus formation assays also demonstrate reduced thrombus formation in the presence of carbenoxolone or probenecid [18,21]. Furthermore, potential roles for platelet gap junctions and pannexin channels in ischemic stroke are not resolved, although a middle cerebral artery occlusion stroke model demonstrated that female Panx1-deficient mice exhibited reduced neurological impairment at 48 h compared with controls [59]. These data suggest that there may be therapeutic potential in repurposing drugs that are well-tolerated, for the cost-effective management of thrombosis and ischemic stroke.

## Future Directions and Conclusions

To date, studies have primarily focussed on the role of platelet connexins and pannexins, however, these proteins are also expressed within the megakaryocyte. Within the bone marrow, Cx43 is the predominant connexin isoform and is expressed by megakaryocytes [60]. Indeed, megakaryocytes have been shown to reduce deposition of type I collagen by osteoclasts, through Cx43-mediated interactions [61]. The direct roles of connexins and pannexins during megakaryocyte development and thrombopoiesis have not been evaluated, but data suggest that platelet counts are normal in both Cx37 and panneixn-1 knockout mice [5,25].

Despite their structural and functional similarities, there is relatively little data available on the interactions between the pannexins and connexins. Noveielli-Kuntz and colleagues evaluated the cardiovascular phenotype of Cx40^−/-^, Panx1^−/-^ and Cx40^−/-^Panx1^−/-^ mice [62]. Cx40-deficient mice have impaired vasodilation of aortic segments, leading to hypertension, a phenotype not observed in Panx1^−/-^ mice. This phenotype was explored further with the double knockout and Panx1 depletion could not rescue the vascular phenotype of Cx40^−/-^ [62]. However, the impact of this model upon platelet function and thrombus formation was not assessed. *In vivo* thrombosis studies with Cx40^−/-^ [6] and Panx1^−/-^ [25] mice both report reduced thrombus formation, indicating therapeutic potential in targeting these channels.

Platelets express Panx1 and multiple members of the connexin gap junction family. These channels are activated following platelet stimulation, contributing to ATP release, Ca[2]+ signaling and platelet activation dynamics. Our understanding of the role for platelet gap junctions during thrombus formation is currently limited. Gap junctions may represent, however, a route for rapid transmission of signals between the core and shell of a growing thrombus. Finally, these channels have been shown to be susceptible to drugs that are in clinical use, which could potentially be repurposed to manage arterial thrombosis.
